# HIV prevalence correlated with circumcision prevalence and high-risk sexual behavior in India's states: an ecological study

**DOI:** 10.12688/f1000research.17807.2

**Published:** 2019-06-17

**Authors:** Chris R. Kenyon

**Affiliations:** 1 Department of Clinical Sciences, Institute of Tropical Medicine, Antwerp, Antwerp, 2000, Belgium; 2 University of Cape Town, Cape Town, South Africa, 7925, South Africa

**Keywords:** India, HIV prevalence, high-risk sex, sexual network, ecological, circumcision

## Abstract

**Background:** HIV prevalence varies between 0% and 1.6% in India's states. The factors underpinning this variation are poorly defined.

**Methods:** We evaluated the relationship between HIV prevalence by state and a range of risk factors in the Indian 2015 National Family Health Survey. Pearson’s correlation was used to assess the relationship between HIV prevalence and each variable. The prevalence of each risk factor was compared between five high-HIV-prevalence states (>1% prevalence) and a large low-HIV-prevalence state (Uttar Pradesh; HIV prevalence, 0.06%).

**Results:** There was an association between HIV prevalence and men's mean lifetime number of partners (r = 0.55; P = 0.001) and men reporting sex with a non-married, non-cohabiting partner (r = 0.40; P = 0.014). In general, men in high-prevalence states were less likely to be circumcised and (with the exception of Chandigarh) use condoms at last sex. In two high prevalence states (Mizoram and Nagaland), men reported a higher number of lifetime partners and a higher prevalence of multiple partners and high-risk sex in the past year.

**Conclusions:** Variation in circumcision prevalence and sexual behavior may contribute to the large variations in HIV prevalence by state in India.

## Introduction

There is little consensus as to whether or not differences in sexual behavior play an important role in determining the large differences in HIV prevalence between populations. Certain authors have claimed that non-behavioral factors such as differences in prevalence of herpes simplex virus 2 infection, circumcision rates and STI treatment efficacy are responsible for differences in HIV prevalence ^1, 2^. Other authors have found that HIV prevalence is associated with high-risk sexual behavior ^3– 5^. These latter studies have included ecological-level analyses. The rationale behind ecological studies is that the prevalence of sexually transmitted infections (STIs) is, to an important extent, determined by the structure of the local sex network ^6^. This is a population-level characteristic and thus ecological studies are required to assess if correlates of this network structure are associated with HIV prevalence ^7^. A number of studies ^3, 5, 8, 9^, but not all studies ^10^, have found that correlates of network connectivity, such as rate of partner change and the prevalence of sexual partner concurrency, are positively associated with HIV prevalence. These associations have been found to be strongest when comparing ethnic groups and regions within countries ^3, 5, 8, 9, 11, 12^. Nationally representative, HIV-serolinked Demographic Health Surveys (DHS) have been a particularly useful resource for these studies ^3, 8, 11^.

India is an interesting country to test the network connectivity thesis. HIV was first detected in India in 1986 and since then a range of sources have documented higher HIV prevalence in certain North East States and to a lesser extent Karnataka, Andhra Pradesh and surrounding areas ^13– 17^. Individual-level analyses have found that well-established risk factors, such as partner number, contact with sex workers, intravenous drug usage and lack of circumcision, were associated with HIV infection ^13, 17– 20^. These analyses have, however, not explained the differential HIV prevalence by state. A number of authors have argued that the higher HIV prevalence in Northeast India may be due to patterns of intravenous drug use (IVDU) in this region. Differences in contact with sex workers, patterns of migration, gender inequality and non-traditional forms of sex work, such as *devadasi* in Karnataka (where women are 'married' to temple deities and have sex with temple attendees), are some of the other factors that have been advanced as reasons for differences in HIV spread in India, often with little supporting evidence ^13, 16– 20^. In this analysis we address this issue by assessing whether there is an ecological-state-level association between various HIV risk factors and HIV prevalence.

## Methods

**Figure 1.**
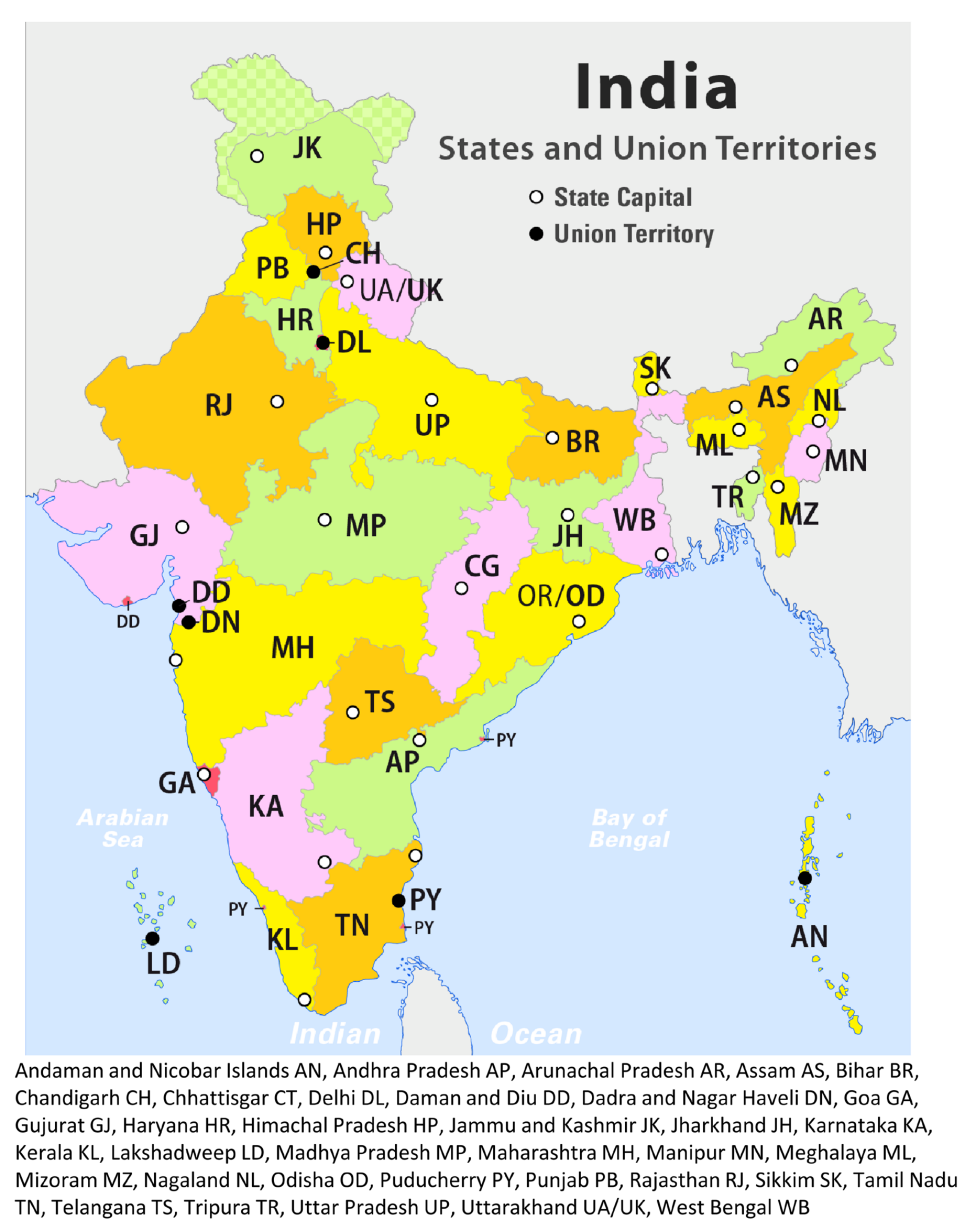
Map of India's 29 states and 7 union territories. Reprinted from 21 under a CC BY license, with permission from Dörrbecker, original copyright 2005.

India is a federal union comprising 29 states and 7 union territories ( Figure 1). We refer to these 36 entities as states. The states are typically large and vary in surface area from Rajastan’s 342,239km ^2^ to Goa’s 3,702km ^2^ (median 88,752km ^2^). The Union Territories are far smaller (median size 491km ^2^) and for this reason are indicated with circles on the map in Figure 1. India’s vast size incorporates a wide range of ethnic and liguistic groups. It has an estimated 2000 ethnic groups and classifies 23 languages as official ^15^. There are considerable differences between states in ethnic composition as well as in education al attainment, life expectancy and poverty rates ^14, 15^.

## Data source

We used the 2015 National Family Health Survey (NFHS-4) for this study. The NHFS-4 received ethical committee clearance for data analyses such as the one performed here. As a result, no specific ethics committee approval was necessary for this study.

The NFHS-4 used a household-based, two-stage stratified sampling approach. The first stage selected primary sampling units (PSUs) from the 2011 National Census. PSUs were villages in rural areas and census enumeration blocks in urban areas. In the second stage of the survey, 22 households were randomly selected with systematic sampling from every selected rural and urban cluster.

A total of 98% of selected households were successfully interviewed. In the interviewed households, women aged 15–49 and men aged 15–54 were eligible to participate. The individual response rate for women was 97% and 92% for men. The response rates were high in all states except for Delhi and Chandigarh ( Table 1). The behavioral questionnaire was administered in 17 local languages using computer-assisted personal interviewing. Further details of the survey are provided in Table 1 and the NFHS-4 report ^14^.

**Table 1.** Prevalence of HIV and associated risk factors in 15–49-year-old women and 15–54-year-old men by state in India in 2015 (arranged according to descending HIV prevalence).

State	N	Age Men & Women ^a^	Age Men ^a^	Age Women ^a^	HIV Total N tested	HIV (%)	HIV Testing Rate (%)	Individual Response Rate Men (%)	Individual Response Rate Women (%)	Circumcision (%)	Condom Men (%)	Condom Women (%)	High risk sex Men (%)	High risk sex Women (%)	Multiple partners Men (%)	Multiple partners Women (%)	Lifetime sex partners Men ^e^	Lifetime sex partners Women ^e^	90+ Partners Men (%) ^b^	90+ Partners Women (%) ^b^	% attained no or only primary education ^c^	% in the Poorest Wealth Quintile ^d^
Mizoram	2667	32.9	31.9	34.9	2607	1.6	97.1	95.7	98.3	2.5	2.9	9.1	20.0	0.0	3.8	0.1	3.4	1.1	0.1	0.2	20.2	5.1
Nagaland	2410	33.1	32.6	34.1	2122	1.4	85.2	92.3	95.8	4.3	3.1	10.5	14.9	0.2	1.0	0.2	1.7	1.2	0.0	0.6	28.2	11.1
Andhra Pradesh	2524	33.4	33.5	33.3	2330	1.2	85.5	85.6	93.8	10.6	0.6	1.9	2.8	0.0	1.1	0.0	0.9	1.0	0.0	0.4	41.1	3.6
Manipur	2935	33.1	32.4	34.3	2889	1.2	97	94.1	97.1	6.8	3.3	7.7	7.0	0.0	0.9	0.0	0.8	1.0	0.0	0.1	19.1	9.8
Chandigarh	194	32.9	31.6	35.5	178	1.1	78.3	78.4	86.8	7.0	38.0	25.5	13.1	0.0	0.0	0.0	0.8	1.0	0.0	0.0	18.0	0.0
Delhi	982	30.0	29.3	32.6	697	0.8	55.5	52.9	82.2	21.8	22.5	32.2	5.6	0.0	0.4	0.2	0.8	1.0	0.0	2.2	24.5	0.6
Daman and Diu	631	29.5	28.9	31.4	561	0.7	86.4	90.9	94.6	7.2	9.0	12.9	4.7	0.0	0.0	0.0	0.6	1.1	0.0	7.5	20.3	0.0
Karnataka	6333	32.6	32.8	32.2	6033	0.6	90.9	89.7	94.5	18.1	7.7	7.7	4.2	0.4	1.7	3.7	0.7	1.2	0.3	0.5	32.5	6.0
Telangana	1813	32.0	32.0	31.9	1677	0.6	81.2	82.6	91.8	15.1	2.0	3.9	5.8	0.6	2.2	0.8	0.9	1.0	0.3	1.4	39.4	5.5
Maharashtra	7546	31.9	31.6	32.3	7103	0.4	89.2	89.2	94.3	14.8	10.5	19.2	7.4	0.2	1.1	0.3	0.8	1.0	0.4	1.0	23.6	8.9
Bihar	9343	31.3	30.8	32.1	9175	0.2	97	96.3	98.4	16.6	4.4	7.0	4.1	0.3	1.5	0.8	0.9	1.2	0.3	1.0	45.2	42.0
Gujarat	9478	32.5	32.0	33.6	9028	0.2	90.5	88.3	94.9	9.0	6.9	8.9	6.4	0.3	1.3	0.3	1.1	1.1	0.8	0.7	31.8	8.8
Chhattisgarh	6147	32.1	31.6	32.9	6030	0.2	96.2	94.1	97.5	4.8	5.7	10.5	9.6	0.1	1.4	0.1	1.0	1.1	0.2	0.3	40.5	30.6
Haryana	5700	31.3	30.8	32.1	5512	0.2	96.2	97	98.9	7.8	17.4	18.4	8.7	0.6	1.9	0.7	0.9	1.0	0.2	0.0	25.1	1.5
Tamil Nadu	8556	33.3	33.3	33.3	8338	0.2	96.4	96.1	98.5	16.9	6.2	6.0	4.4	0.1	1.5	0.6	0.8	1.0	0.2	0.3	25.2	2.8
Uttarakhand	3333	31.8	31.1	33.3	3203	0.2	95.3	92.4	97.2	14.1	21.8	20.1	6.9	0.6	1.7	0.5	0.7	1.0	0.3	0.8	26.9	4.3
Punjab	5024	32.3	31.6	33.5	4932	0.2	95.1	93.4	97.8	3.7	22.0	21.7	14.7	0.8	1.8	0.7	0.9	1.0	0.2	0.2	25.2	1.2
Madhya Pradesh	16412	31.9	31.4	32.7	15964	0.1	96	95.6	97.7	8.8	9.3	14.3	10.3	0.3	2.2	0.5	1.1	1.1	0.7	0.5	44.7	28.1
Rajasthan	9949	31.3	30.6	32.6	9787	0.1	96.6	95.2	97.9	8.2	11.1	11.1	5.8	0.1	1.0	0.1	0.8	1.0	0.1	0.7	40.9	15.6
Assam	6682	32.2	32.5	31.6	6369	0.1	92.2	90.1	96.1	28.2	6.3	9.1	1.7	0.2	0.6	0.2	0.7	1.0	0.3	0.5	34.7	21.9
West Bengal	4297	32.5	33.1	31.5	4111	0.1	93	93	97.1	24.7	8.0	11.1	3.3	0.5	0.8	0.5	0.9	1.0	0.2	0.1	39.7	22.5
Jammu and Kashmir	9225	32.5	31.7	34.1	8945	0.1	95.3	92.2	97.4	63.7	15.0	15.9	2.2	0.0	0.4	0.3	0.7	1.1	0.2	0.3	31.4	8.5
Himachal Pradesh	3719	33.5	32.8	34.8	3548	0.1	91.7	84.4	95.4	4.3	14.8	21.7	6.1	0.1	1.1	0.3	1.1	1.0	0.1	0.0	18.6	1.5
Jharkhand	6486	31.3	31.1	31.7	6229	0.1	92.9	90.1	95.4	17.4	5.4	4.8	4.5	0.3	1.0	0.5	0.9	1.1	0.2	1.4	40.0	43.7
Uttar Pradesh	21001	31.3	30.4	33.0	20569	0.1	91.9	86.9	96.1	19.1	13.1	14.5	8.5	0.2	1.5	0.3	1.0	1.1	0.2	1.1	39.5	23.1
Arunachal Pradesh	3374	32.6	32.6	32.7	3100	0.1	87.1	88.6	93.3	5.1	8.1	13.4	14.3	0.2	2.8	0.4	1.1	1.1	0.9	1.0	40.3	17.6
Meghalaya	1904	31.5	31.0	32.5	1823	0.1	94.1	91.1	96.9	7.8	4.5	5.9	5.7	0.3	0.9	0.0	0.8	1.1	0.0	0.0	41.0	9.9
Sikkim	1374	33.1	33.0	33.3	1364	0.0	97.4	97.3	98.1	3.1	9.3	18.0	13.1	0.0	1.0	0.0	1.2	1.0	0.1	0.0	32.9	0.4
Puducherry	1098	33.7	33.9	33.3	1095	0.0	98.6	96.5	99.4	20.0	3.3	9.5	1.5	0.0	0.6	0.0	0.7	1.0	0.0	0.2	16.6	1.3
Odisha	7514	33.1	33.2	32.9	7197	0.0	93	91.2	96.8	4.2	11.4	13.2	1.8	0.1	0.7	0.5	0.9	1.0	3.8	1.5	38.5	32.8
Andaman and Nicobar islands	705	34.5	34.8	34.1	295	0.0	96	85.3	94.2	10.4	7.6	16.2	9.9	1.7	2.8	1.5	1.1	1.0	0.5	0.0	23.5	4.3
Dadra and Nagar Haveli	322	30.5	29.9	31.8	690	0.0	94.1	93.5	97.6	17.5	1.3	16.8	6.3	0.0	1.8	0.0	0.8	1.0	0.0	0.0	35.4	20.2
Kerala	3189	34.0	33.5	34.8	229	0.0	98.3	96.6	97.6	28.7	5.0	12.4	3.6	0.2	1.0	0.2	0.8	1.0	0.0	0.0	7.1	0.6
Lakshadweep	241	34.6	34.2	35.4	1282	0.0	92	97.4	98.8	94.5	10.4	5.3	4.7	0.0	0.6	0.0	0.6	1.0	0.0	0.0	8.7	0.0
Goa	1291	34.6	34.4	35.0	3066	0.0	94	95.1	98.3	7.6	15.4	30.1	10.8	0.2	0.9	0.3	0.8	1.2	0.1	0.8	13.9	0.0
Tripura	1419	32.4	32.7	31.9	1343	0.0	91.9	89.2	96.4	2.4	4.4	4.8	1.8	0.0	0.4	0.0	0.7	1.0	0.6	0.0	29.5	14.4

^a^Average age in years by state.

^b^ "90+ Partners" – This variable refers to the percent per state reporting more than 90 lifetime sex partners (for men and women) separately, of all respondents.

^c^ Highest education level attained: Percent whose highest educational attainment is primary on no schooling.

^d^ Percent in the Poorest Wealth Quintile: this variable refers to the percent of the respondents from this region that were calculated to fall in the poorest 20% (quintile) of the nationally derived wealth band. The wealth quintiles were derived from an asset index.

^**e**^ Lifetime number of partners refers to the mean number of lifetime partners reported by those who reported being sexually experienced, excluding those with more than 90 partners.

## Measures

### HIV Testing and Prevalence estimates

In a random subsample of households, a dried blood spot from a finger-prick blood specimen was obtained from eligible women age 15–49 and men age 15–54 who consented to laboratory HIV testing. Respondents did not have access to the test results but were all referred to counseling and testing services in the local area. Dried blood samples were collected and subsequently tested for HIV using a Microlisa ELISA. Positive tests and a random 2% sample of negative samples were then tested with SD Bioline 1/2 ELISA (Standard Diagnostics Inc., Kyonggi-do, South Korea). Discordance between the two tests was resolved with Western Blot (BioRad). With the exception of Delhi, HIV testing rates were high in all states (median 95.9%, IQR 94.4-97.9%). Coverage was higher in women (95%) and men (90%) in rural areas as well as urban areas (women, 91%; men, 84%).

The NFHS-4 was designed to provide HIV prevalence estimates that are representative for the women (15–49 years) and men (15–54 years) for the whole country and rural and urban areas. It also oversampled certain states so as to be able to generate representative samples for 11 groups of states ( Table 2). These were states that had been found to have higher HIV prevalence rates in previous surveys, as well as the Uttar Pradesh-centered group of states, which was chosen as a low-HIV-prevalence comparator. These 11 groups of states consisted of composites of vast populations with considerable heterogeneity in sexual behaviors and circumcision prevalence ( Table 1). Our primary research question involved assessing if HIV-related risk factors differed between high- and low-HIV-prevalence states. To avoid problems related to averaging out large differences in prevalence of risk factors in the groups of states, in our primary analysis we compared risk factors with estimated HIV prevalence by state. In a sensitivity analysis this analysis was repeated using the 11 groups of states.

***High HIV prevalence states***. UNAIDS defines populations with a 15-49-year-old HIV prevalence of greater than 1% as a generalized HIV epidemic ^22^. We therefore defined states with a 15-49-year-old HIV prevalence of above 1% as high-HIV-prevalence states.

***Low HIV prevalence comparator state***. Uttar Pradesh was chosen as the low-HIV-prevalence comparator state for a number of reasons. The previous HIV-serolinked NHFS, as well as the current NFHS survey, found an adult HIV prevalence of less than 0.1% in Uttar Pradesh ^15^. It also has the largest population of any state in India and as a result it had the largest sample size of all states in NFHS-4. In sensitivity analyses we repeated the analyses using all Indian states with a HIV prevalence below 0.2% as the low HIV prevalence comparator population.

All HIV-prevalences were calculated for 15–49-year-olds.

### Independent variables

Each of following predictor variables were calculated separately for men and women and were limited to those between the ages of 15–49 years for women and 15–54 years for men.

- Condom use at last sex: The percentage of respondents who reported using a condom at last sex, amongst those who have had sex in the past 12 months.
- Male circumcision: The percentage of men who reported being circumcised.
- High-risk sex (sex with a non-married, non-cohabiting partner): The percentage of respondents who reported sex with a non-marital, non-cohabiting partner in the past 12 months, amongst all respondents who reported sex in the past 12 months.
- Multiple partners in the past year: The percentage with two or more sexual partners in the past 12 months amongst all respondents who reported sex in the past 12 months.
- Lifetime sex partners: The mean number of reported lifetime sex partners amongst all respondents who reported having had sex, excluding those with greater than 90 partners.

The reason for excluding those with 90 or more partners was that in a small number of states these individuals exerted a large effect on the mean number of lifetime partners. Our primary research question was whether or not there were population differences in lifetime number of partners for the majority of the population. The percent of the population with 90 partners or more was small in all states: men, median 0.17 (IQR 0-0.29); women, median 0.39 (IQR 0.2-0.89) ( Table 1). As a result we found it more informative to calculate mean lifetime number of partners excluding those with more than 90 partners.

Two socioeconomic control variables were assessed:

- Education attained: Percent of state respondents whose highest educational attainment is primary or no schooling.
- Poverty: Percentage of the respondents from this state that were calculated to fall in the poorest 20% (quintile) of the nationally derived wealth band. The wealth quintiles were derived from an asset index.

## Statistical analysis

All analyses are ecological in nature and conducted with HIV prevalence by state or group of states as the outcome variable. The analyses were conducted using STATA 13.0 (College Station, TX) and were all adjusted to account for the complex sampling strategies of the survey using the survey (SVY) command. The analyses were stratified by gender. The two-sample Wilcoxon rank-sum test was used to assess if there was a difference in the median number of lifetime partners between the high-HIV-prevalence states and Uttar Pradesh/low-HIV-prevalence states. Cohen's d was used to assess effect size. Histograms were used to depict the distribution of the number of lifetime sexual partners by gender and state. Chi-squared tests were used to assess differences in categorical variables. Pearson’s correlation was used to assess the relationship between state-level HIV prevalence and the prevalence of each risk factor.

## Results

### Demographic variables and HIV prevalence

An overview of the sample size, mean ages and other demographic characteristics of men and women by state are provided in Table 1. The average age of respondents in the regions ranged from 28.9 to 34.8 years in men (median 32.0 [IQR 31.1-33.0]) and 31.4 to 35.5 years in women (median 33.0 [IQR 31.4-35.5]).

The overall 2015 HIV prevalence in 15–49-year-olds was 0.25% in men and 0.23% in women. The median HIV prevalence by state was 0.13% (IQR 0.05-0.48%) and the prevalence in Uttar Pradesh was 0.06% ( Table 1).

The prevalence of HIV exceeded 1% in 5 states. Three of these were in the North East (Manipur, 1.2%; Nagaland, 1.4%; and Mizoram, 1.6%) and two elsewhere (Andhra Pradesh, 1.2%; and Chandigarh, 1.1%). India’s prior HIV prevalence survey (NFHS-3) in 2005–2006 was designed to provide HIV prevalence estimates from six states ^15^. Of these, Manipur had the highest HIV prevalence (1.13%), followed by Andra Pradesh (0.97%), Karnataka (0.69%), Maharashtra (0.62%), Tamil Nadu (0.34%) and Uttar Pradesh (0.07%) in 15–49 year old men and women.

### Comparison of risk factors between high and low HIV prevalence states

***Circumcision***. The prevalence of circumcision was low in all states, including Uttar Pradesh (19.1%), but was significantly lower in all the high-HIV-prevalence states (2.5–10.6%; all P<0.0005).

***Condoms at last sex***. Men/women in Andhra Pradesh (1.9/0.6%), Manipur (7.7/3.3%), Mizoram (9.1/2.9%) and Nagaland (10.5/3.1) were less likely, but those from Chandigarh (25.5/38.0%) were more likely to report using a condom at last sex than those from Uttar Pradesh (13.5/13.1%; all P-values <0.05).

***High-risk sex***. The prevalence of reported high-risk sex in men was 8.3% in Uttar Pradesh. A higher proportion of men in Mizoram (20%) and Nagaland (15%) but a lower proportion in Andhra Pradesh (2.8%) reported higher risk sex (all P<0.0005). Reported proportions in women were low and did not differ between states.

***Multiple partners in past year***. Men in Mizoram were more likely to report multiple partners (3.8%) in the past 12 months than Uttar Pradesh (1.5%) (P<0.005).

**Figure 2.**
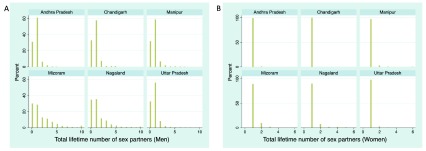
Histograms of the reported number of lifetime sexual partners by state. ( **A**) men aged 15–54 years and ( **B**) women aged 15–49 years. Data from five high HIV prevalence states and Uttar Pradesh. Distributions are truncated at 10/6 partners for men/women.

***Lifetime partners***. Men/women in Mizoram (3.39/1.12) and Nagaland (1.74/1.15) reported a higher number of lifetime partners than Uttar Pradesh (0.97/1.07); women in Andhra Pradesh reported fewer partners (1.01) (all P<0.0005). The magnitude of the right shifting in the distribution of lifetime number of partners was large for men in Mizoram and Nagaland (Cohen's d = 0.68 and 0.43, respectively) and small elsewhere ( Table 2; Figure 2).

Two sensitivity analyses were performed. Firstly, repeating the analyses using all Indian states with HIV prevalences below 0.2% as the low-HIV-prevalence comparator population (instead of Uttar Pradesh) had little effect on the results ( Table 2). Secondly, repeating the analyses using the 11 groups of states in place of the 36 states produced comparable results ( Table 3). Low circumcision and condom-usage rates together with lifetime number of partners and high-risk sex (men only) remained significant risk factors in group 7 states (Mizoram, Manipur, and Nagaland) compared to the Uttar Pradesh group (Uttar Pradesh, Madhya Pradesh, Uttarakhand, and Rajasthan).

**Table 2.** Comparison of prevalence of HIV-related risk factors in high HIV prevalence states (>1%; (Andhra Pradesh, Chandigarh, (Manipur, Mizoram and Nagaland) with Uttar Pradesh (A) and all low HIV prevalence states (≤0.2%; B).

	HIV prevalence (%)	Circumcision (%)	High-risk sex (%)		Lifetime number of sex partners (mean)		Multiple partners past year (%)		Condom use at last sex (%)	
		Men	Women	Men	Women	Men	Women	Men	Women	Men
High HIV Prevalence States										
Andhra Pradesh	1.21	10.6 ***	0.0	2.8 ***	1.01 **	0.92	0.0	1.1	0.6 ***	1.9 ***
Chandigarh	1.1	7.0 **	0.0	13.1 *	1.00	0.80	0.0	0.0	38.0 ***	25.5 *
Manipur	1.17	6.8 ***	0.0	7.0	1.03	0.84	0.0	0.9	3.3 ***	7.7 ***
Mizoram	1.62	2.5 ***	0.0	20.0 ***	1.12 ***	3.39 ***	0.01	3.8 **	2.9 ***	9.1 ***
Nagaland	1.39	4.3 ***	0.2	15.0 ***	1.15 ***	1.74 ***	0.02	1.0	3.1 ***	10.5
Low HIV Prevalence States										
A) Uttar Pradesh (Low HIV ref)	0.06	19.1	0.2	8.3	1.07	0.97	Ref		0.03	1.5
B) All states with HIV prevalence ≤0.2%	0.12	16.2	0.25	6.2	1.07	0.92	0.4	1.3	9.1	11.5

^*^P<0.05 ^**^P<0.005 ^***^P<0.0005

**Table 3.** Comparison of HIV-related risk factors in 11 groups of states in NFHS-4. P-values are for comparisons between group 11 (low HIV prevalence group) and groups 1 and 7 (high-HIV-prevalence groups).

Groups	HIV (%)	Lifetime partners (mean)		High-risk sex (%)		Multiple partners in past year (%)		Circumcision (%)	Condom (%)	
	Women and Men	Women	Men	Women	Men	Women	Men	Men	Women	Men
Group 1: Andhra Pradesh and Telangana	0.91	1.01 ***	0.92 **	0.2	4.0 **	0.3	1.5	12.4 *	1.1 ***	2.7 ***
Group 2: Bihar, Jharkhand, West Bengal, and Andaman & Nicobar Islands	0.15	1.11	0.88	0.4	3.8	0.6	1.1	20.5	6.3	8.7
Group 3: Gujarat, Dadra & Nagar Haveli, and Daman & Diu	0.18	1.09	1.14	0.3	6.4	0.3	1.3	9.1	6.9	9.0
Group 4: Himachal Pradesh and Jammu & Kashmir	0.08	1.06	0.83	0.0	3.7	0.3	0.6	42.4	15.0	18.2
Group 5: Karnataka	0.64	1.18	0.69	0.4	4.2	3.7	1.7	18.1	7.7	7.7
Group 6: Maharashtra and Goa	0.39	1.04	0.85	0.2	7.6	0.3	1.1	14.6	10.7	19.6
Group 7: Mizoram, Manipur, and Nagaland	1.49	1.08 ***	1.65 ***	0.1	12.1 ***	0.1	1.6	5.0 ***	3.1 ***	8.8 ***
Group 8: Odisha and Chhattisgarh	0.13	1.05	0.93	0.1	5.5	0.3	1.0	4.4	8.9	11.9
Group 9: Punjab, Haryana, Delhi, and Chandigarh	0.25	1.03	0.87	0.6	10.7	0.6	1.5	9.9	20.3	22.3
Group 10: Tamil Nadu, Kerala, Puducherry, and Lakshadweep	0.17	1.04	0.78	0.2	4.2	0.5	1.3	20.2	5.9	7.8
Group 11: Uttar Pradesh, Madhya Pradesh, Uttarakhand, and Rajasthan	0.10	1.06	0.97	0.2	8.4	0.3	1.5	14.3	11.9	13.9

^*^P<0.05 ^**^P<0.005 ^***^P<0.0005.

### State-level correlates of HIV prevalence

Controlling for education and poverty levels, the prevalence of higher-risk sex in men (r=0.40; P=0.017) and mean number of lifetime partners in men (r=0.55; P=0.001) were positively associated with HIV prevalence ( Table 4).

**Table 4.** Pearson’s correlation coefficients for the association between HIV prevalence and various risk factors by region in India's 36 States in NFHS-4.

Variable	Men	Women
Circumcision	-0.23	NA
Condom usage	-0.007	-0.07
High-risk sex	0.40 *	-0.22
Multiple partners past year	0.11	-0.03
Mean no. of lifetime sex partners	0.55 **	0.12
Education attained	-0.14 †	-0.14 †
Poverty	-0.24 †	-0.24 †

^*^ P<0.05, ^**^ P<0.005. † These two variables were calculated with the combined men and women's education/poverty data and hence are the same for men and women. NA, not applicable.

## Discussion

As in other countries, the spread of HIV has been heterogenous in India. Whilst the absolute differences in HIV prevalence by state are not large, the relative differences are. A state-level HIV prevalence above 1% is four times the national average prevalence. These differences in HIV prevalence are also fairly stable based on both data from the 2005 NFHS survey ^15^ and other data sources such as antenatal surveys from the 2000s until the present ^13, 16^. Previous studies have argued that the higher prevalence rates in certain states in Northeast India is predominantly due to patterns of IVDU ^13, 16^. We were unable to investigate the role of IVDU, but we found population-level differences in the prevalence of circumcision, condom usage and sexual behaviors, which could explain differential HIV spread.

The most consistent association we found was lower circumcision prevalence rates in the high HIV prevalence states. Circumcision rates were, however, as low or lower in a number of low HIV prevalence states as in the high HIV prevalence states. Although differences in condom usage were statistically significant, the absolute differences were small and one high-HIV-prevalence state had higher condom utilization than the low-HIV-prevalence comparators.

The relationship between the sexual behavioral risk factors and HIV prevalence was not uniform. Whilst sexual behaviors were in general riskier in the North East states, this was not the case in Andhra Pradesh and Chandigarh, where a number of the behavioural risk factors were actually less prevalent than Uttar Pradesh. In a country as vast and diverse as India, it should not be too surprising to find different combinations of risk factors to be responsible for HIV spread in different regions. As noted above, previous studies have suggested that various factors in Andhra Pradesh and Karnataka may play a role in the local HIV epidemics here ^13, 20^. An important finding of our study was the higher number of lifetime partners, multiple partners in the prior year and high-risk sex in Mizoram and Nagaland compared to Uttar Pradesh. Our study is thus compatible with the thesis that these behaviors would translate into a more connected sexual network which could play a role in the generation of higher HIV prevalence rates in these states.

These same three risk factors (number of lifetime partners, multiple partners in the prior year and high-risk sex) have previously been found to be associated with differences in HIV prevalence by ethnic group in Kenya ^3^, South Africa ^5^ and elsewhere ^8, 23^. Previous reports from DHS data found that, at an individual level, there was a stepwise increase in HIV prevalence with increasing lifetime sex partners in all 15 countries with available data. This was true for both men and women in all cases ^4, 24^. This association was also present in the Indian NFHS-3 in 2005 and in this survey ^14, 15^. That this association was present at both individual and population levels increases the probability that the association between lifetime sex partners and HIV prevalence is real ^25, 26^. In our study, this association was, however, only statistically significant for men. High-risk sex has also been found to be an individual level risk factor for HIV infection in a number of a number of countries, including India ^4, 14^. As in other areas, it is likely that these patterns of higher risk sexual behavior interact with other risk factors such as IVDU to produce the observed differences in HIV prevalence ^16, 19^.

This study has a number of limitations. The study is ecological in nature and is thus susceptible to the ecological inference fallacy. DHS surveys are not optimal for collecting sensitive sexual information ^27, 28^. The use of computer-assisted interviewing would be expected to have reduced but not eliminated this problem ^27^. In addition, although the response rates for participation in the survey and HIV testing were high, this varied somewhat between states. The survey was designed to provide HIV prevalence estimates for 11 groups of states and not individual states. The data is thus susceptible to a large number of biases such as misclassification, nonresponse, recall and social desirability biases. In particular, other work has found evidence of culture-specific heterogeneity in answering questionnaires ^29, 30^. We cannot exclude the possibility that respondents from states where lower-risk sexual behavior was reported were subject to a greater social desirability bias, which could invalidate our findings. The available evidence, however, suggests that only minor differences in sexual behavior exist between those who do and do not answer sexual behavior questionnaires ^31^. Finally, the analyses do not control for the influence of other variables.

## Conclusion

We found a range of risk factors to be more prevalent in high-HIV-prevalence states in India. There was no clear single risk factor (or combination thereof) which appeared capable of explaining the heterogeneity of HIV spread in India. In the case of Mizoram and Nagaland, however, a higher prevalence of sexual risk behaviors may be contributing to higher HIV prevalence rates in this region. More detailed comparative studies between these populations and lower prevalence populations elsewhere in India may confirm or refute this finding.

Studies of other higher-HIV-prevalence-populations that have managed to reduce HIV incidence, including Uganda, Zimbabwe, Kenya and Thailand, have pointed to the importance of reductions in multiple partnering in effecting this decline ^32– 35^. If other studies confirm our findings, then similar campaigns could be considered in Mizoram and Nagaland.

## Data availability

The NFHS-4 survey is freely available from www.measureDHS.com as part of the India: Standard DHS, 2015–16. Access to the dataset requires registration, and is granted to those that wish to use the data for legitimate research purposes. A guide for how to apply for dataset access is available at: https://dhsprogram.com/data/Access-Instructions.cfm.
