# Supplementary material for: HIV prevalence correlated with circumcision prevalence and high-risk sexual behavior in India's states: an ecological study
Source: F1000Res. 2019 Jun 17;8:60. Originally published 2019 Jan 15. [Version 2] doi: 10.12688/f1000research.17807.2 (PMC6611127; doi:10.12688/f1000research.17807.2)
Supplement: Peer review history

# Peer review: Reviewer response for version 2

**DOI:** 10.5256/f1000research.21562.r49983

**Published:** 2019-07-04

**Authors:** Pauline E. Jolly

**Affiliations:** 1 Department of Epidemiology, University of Alabama at Birmingham, Birmingham, AL, USA

I have gone over the paper and the revisions have been made. The paper is now acceptable for indexing. There is a typographical error in the first paragraph of the second column on page 3 (line 8 "education al").

I confirm that I have read this submission and believe that I have an appropriate level of expertise to confirm that it is of an acceptable scientific standard.

# Peer review: Reviewer response for version 1

**DOI:** 10.5256/f1000research.19468.r48166

**Published:** 2019-06-10

**Authors:** John McBride

**Affiliations:** 1 College of Medicine and Dentistry, James Cook University, Cairns, Australia

The paper provides some valuable insights into the transmission of HIV in India. The findings are consistent with other international studies in showing that an increased number of lifetime sexual partners and lower condom use is associated with higher HIV prevalence. It also confirms other ecological studies showing an inverse relationship between circumcision rate and HIV prevalence.

 The data for the study was obtained from the NFHS-4 survey and in the methods section, where you discuss ethics, could you clarify that it is the survey that had ethical approval rather than this study.

 I did have some difficulty following the geography outlined in the paper. In Figure 1, some of the circles for union territories appear not to be associated with any particular area (e.g. DN). There is double labelling for DD and PY and LD is not in the legend. In the text there was mention of 11 groups of states which should have appeared in Table 2 but there are only five states mentioned in this table. Table 4 might be the more appropriate table. Table 2 and Table 3 are almost identical with only the last row different - you could probably get rid of one table by combining them.

 In the discussion, whilst I accept that there is overlap of the circumcision rates between various states with high and low HIV prevalence I don't think there is enough data available in the study to support the contention that circumcision is not an important factor. I think the study supports high-risk sex and condom use as being the most important factors but in states where circumcision rates are low and HIV prevalence high - there should be some consideration of the role of circumcision in the public health response to HIV.

I confirm that I have read this submission and believe that I have an appropriate level of expertise to confirm that it is of an acceptable scientific standard.

# Response

**Published:** 2019-06-12

**Authors:** Chris Kenyon

**Affiliations:** Institute of Tropical Medicine, Antwerp, Belgium

1. The wording as to the ethical approval process has been amended as suggested.
2. The following text has been added to the methods section to assist the reader with understanding the difference in size between Union Territories and States:
  The states are typically large and vary in surface area from Rajastan’s 342,239km ^2^ to Goa’s 3,702km ^2^ (median 88,752km ^2^). The Union Territories are far smaller (median size 491km ^2^) and for this reason are indicated with circles on the map in Figure 1.
  As a result DN is indicated with a circle with no surrounding coloured area.
  The code for ‘LD’ is Lakshadweep and has been added. The double labelling for DD and PY has been rectified.
3. This is a fair comment. To present a more balanced discussion of the role of circumcision, the discussion has been limited to the following:
  The most consistent association we found was lower circumcision prevalence rates in the high HIV prevalence states. Circumcision rates were, however, as low or lower in a number of low HIV prevalence states as in the high HIV prevalence states.

# Peer review: Reviewer response for version 1

**DOI:** 10.5256/f1000research.19468.r45246

**Published:** 2019-03-12

**Authors:** Pauline E. Jolly, Curtis Jolly

**Affiliations:** 1 Department of Agricultural Economics, Auburn University, Auburn, AL, USA; 2 Department of Epidemiology, University of Alabama at Birmingham, Birmingham, AL, USA

1. The study is simple and is a good start for a more complex study with more in-depth analyses.
2. The author should describe the ecological zones in more detail. What makes zones homogenous or heterogeneous (poverty, sexual relations, business activities, etc.)? Can you relate ecological zones with the geographical, economic, ethnic etc., situation in India?
3. The author reports that States were classified as low and high risks previously; it would be important to show a correlation between his/her selected States and the previous high or low risk States.
4. The author states that the study was controlling for two socioeconomic variables (education, and poverty), but never indicated in the results and discussions how the controls affected the results.
5. Simple correlation analyses were conducted, but the author mentions “likelihood” in stating that there is a greater likelihood of having high HIV prevalence if men were uncircumcised or had multiple partners. Greater or lower likelihood cannot be used with simple correlation. We can only talk of a relationship, to report likelihood the author would have to do further statistical analyses such as logi/probit/tobit models, and chance ratio.
6. The results in Tables 3 should be divided between high and lower States. What are the high and low risk States? The signs of the coefficients should be mentioned, what does a positive sign signify? Each table should be fully understandable on its own.

We confirm that we have read this submission and believe that we have an appropriate level of expertise to confirm that it is of an acceptable scientific standard, however we have significant reservations, as outlined above.

# Response

**Published:** 2019-06-12

**Authors:** Chris Kenyon

**Affiliations:** Institute of Tropical Medicine, Antwerp, Belgium

1. Thank you for this comment.
2. We have provided more background information as the heterogeneity of peoples in India’s states (Page 3):
  India is a federal union comprising 29 states and 7 union territories (Figure 1). We refer to these 36 entities as states. The states are typically large and vary in surface area from Rajastan’s 342,239km ^2^ to Goa’s 3,702km ^2^ (median 88,752km ^2^). The Union Territories are far smaller (median size 491km ^2^) and for this reason are indicated with circles on the map in Figure 1. India’s vast size incorporates a wide range of ethnic and liguistic groups. It has an estimated 2000 ethnic groups and classifies 23 languages as official ^15^. There are considerable differences between states in ethnic composition as well as in educational attainment, life expectancy and poverty rates ^14,15^.  
3. This information has been added:
  India’s prior HIV prevalence survey (NFHS-3) in 2005-2006 was designed to provide HIV prevalence estimates from six states ^15^. Of these, Manipur had the highest HIV prevalence (1.13%), followed by Andra Pradesh (0.97%), Karnataka (0.69%), Maharashtra (0.62%), Tamil Nadu (0.34%) and Uttar Pradesh (0.07%) in 15-49 year old men and women.
4. This has been made clear in the new version which now reads (Page 8):
  Controlling for education and poverty levels, the prevalence of higher-risk sex in men (r=0.40; P=0.017) and mean number of lifetime partners in men (r=0.55; P=0.001) were positively associated with HIV prevalence (Table 4).
5. The word ‘likelihood’ has been changed to ‘probability.’
6. Thank you for pointing this out. The five states that are classified as high HIV prevalence are now clearly listed in the title of Table 3. The list of all low HIV prevalence states would be too long to merit inclusion in the title, but the definition of low HIV prevalence state (HIV prevalence ≤0.2%) has been included in the title.
  Table 3. Comparison of prevalence of HIV-related risk factors in high HIV prevalence states (>1%; (Andhra Pradesh, Chandigarh, (Manipur, Mizoram and Nagaland) with low HIV prevalence states (≤0.2%).
  The Table contains prevalence figures of HIV-related risk factors and no coefficients. This has been made clear in the title and in the column headings.
